# Dietary Patterns and the Risk of Composite-Defined Osteoporosis in Pre- and Postmenopausal Women: A Prospective Cohort Study

**DOI:** 10.3390/nu17182947

**Published:** 2025-09-12

**Authors:** Yejung Choi, Kyong Park

**Affiliations:** Department of Food & Nutrition, Yeungnam University, 280 Daehak-ro, Gyeongsan 38541, Republic of Korea; dpwjd0213@yu.ac.kr

**Keywords:** dietary patterns, osteoporosis, menopause, cohort studies

## Abstract

**Background/Objectives**: This study aimed to investigate the association between dietary patterns and the risk of developing osteoporosis in women, emphasizing the role of nutrition in bone health during menopause-related hormonal changes. **Methods**: We conducted a prospective study involving 4865 women aged 40–69 years without osteoporosis at baseline. Dietary information was collected using a validated semi-quantitative food frequency questionnaire, and composite-defined osteoporosis (CDO) was defined using self-administered questionnaires and quantitative ultrasound. Multivariate Cox proportional hazards regression analysis was employed to calculate hazard ratios and 95% confidence intervals to assess the association between dietary patterns and the risk of CDO. **Results**: During a median follow-up of 8.26 years, 895 and 1525 cases of CDO were reported in premenopausal and postmenopausal women, respectively. Three dietary patterns were identified in premenopausal women: the “Vegetables and Seafood,” “Western,” and “White rice, Meat, and Alcohol” patterns. In postmenopausal women, the “Diverse,” “Plant-based,” and “Sweets and Drinks” patterns were identified. Among premenopausal women, the “White rice, Meat, and Alcohol” pattern was associated with a significantly greater risk of CDO in the highest tertile compared with that in the lowest tertile, whereas no significant patterns were observed in postmenopausal women. **Conclusions**: These findings underscore the importance of dietary factors in maintaining bone health, particularly in premenopausal women. Encouraging the reduction in dietary factors associated with an increased osteoporosis risk may help improve bone health and quality of life in women, especially before significant menopause-induced bone loss occurs. This study highlights the need for early dietary interventions to prevent osteoporosis in women.

## 1. Introduction

Osteoporosis, a skeletal disorder, causes damage to bone structure and strength, leading to chronic conditions and various issues, such as falls, fractures, chronic pain, and even premature death [1]. As the global population ages, osteoporosis disproportionately affects women, particularly postmenopausal women, owing to hormonal changes and an increased life expectancy compared to men [2,3]. Globally, one in three women aged over 50 will experience an osteoporotic fracture, representing a significant public health challenge [4,5]. In Korea, the number of patients with osteoporosis increased from 981,000 in 2018 to 1,184,000 in 2022, with approximately 94% of these patients being women [3]. This rise has not only contributed to increased healthcare costs but also imposed significant socioeconomic burdens, as the secondary effects of osteoporosis (e.g., fractures and impaired mobility) diminish quality of life in older women [6,7]. Addressing this issue is requisite to improving women’s health and alleviating long-term societal costs.

The causes of osteoporosis are diverse, including genetic predisposition, physical inactivity, excessive alcohol consumption, smoking, hormonal changes, and nutritional factors [8,9,10,11]. Among these, nutritional factors are exceptionally significant, as they play a crucial role in both the prevention and management of osteoporosis [12]. In women, estrogen loss during and after menopause is a key driver of rapid bone-density loss, rendering nutritional strategies essential for mitigating osteoporosis risk during this life stage [13,14]. Notwithstanding, dietary patterns and their association with osteoporosis risk remain understudied in Asian women, especially when considering menopausal status as a factor.

In recent years, nutritional epidemiology has transitioned from focusing on individual nutrients or foods to exploring overall dietary patterns, which better elucidate the complexity of diet–disease relationships [15,16]. This approach better explains how dietary habits influence osteoporosis risk. For instance, studies have revealed that traditional dietary patterns characterized by an excessive consumption of rice, kimchi, and vegetables, as well as Western dietary patterns rich in sugar, fat, and bread, increase osteoporosis risk in postmenopausal women [17]. Conversely, diets high in fruits and vegetables are associated with lower risks of osteopenia and osteoporosis [18]. Additionally, recent research highlights the benefits of adhering to a Mediterranean diet, which positively affects bone mineral density owing to its emphasis on vegetables, fruits, and fish [19].

However, prior research has primarily focused on postmenopausal women, overlooking the potential influence of premenopausal dietary patterns on postmenopausal osteoporosis risk. Furthermore, several studies have relied on cross-sectional designs, analyzed specific nutrients or food groups rather than comprehensive dietary patterns, and have often failed to incorporate recent data. These gaps accentuate the need for longitudinal studies that examine how premenopausal dietary habits affect bone health during and after the menopausal transition, providing a more holistic understanding of osteoporosis risk across different life stages.

Therefore, this study aimed to examine the association between dietary patterns and osteoporosis risk in Korean women, stratified by menopausal status, using data from a community-based cohort study. It ultimately sought to address existing gaps and provide critical insights for developing nutritional strategies that prevent osteoporosis across key life stages in women.

## 2. Materials and Methods

### 2.1. Study Population

**Figure 1:**
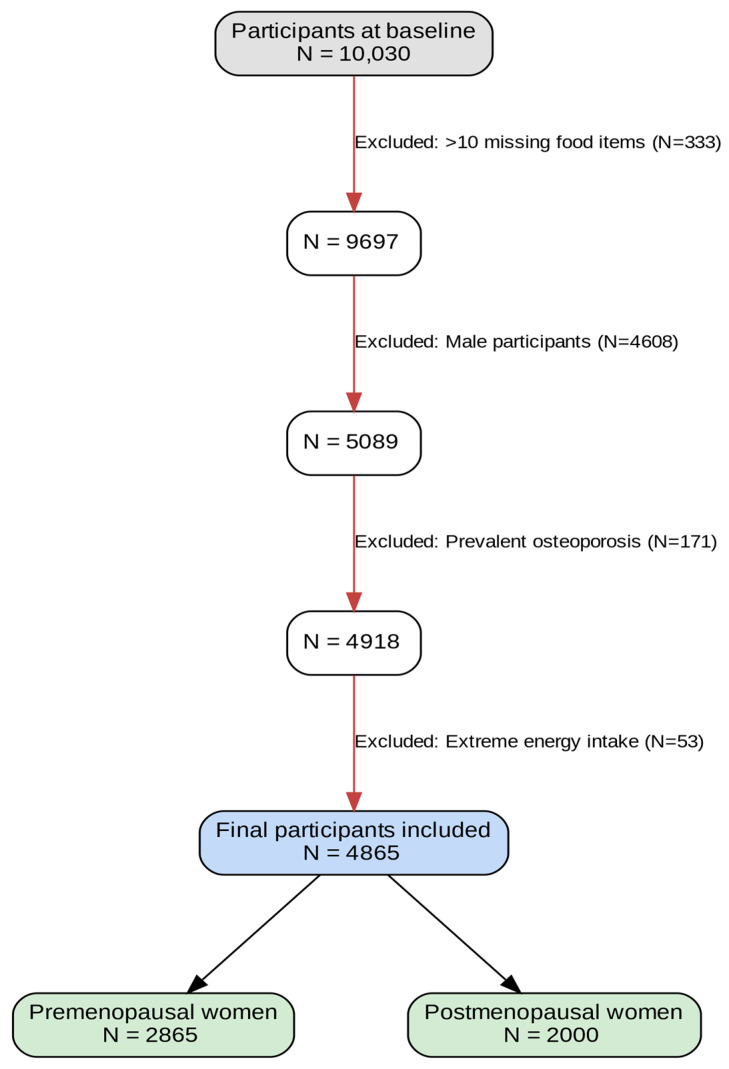
Flowchart of participant exclusion and retention in this study.

This study analyzed data from the Ansan–Ansung cohort of the Korea Association Resource, which is integral to the Korean Genome and Epidemiology Study (KoGES). The Ansan–Ansung cohort is designed to investigate chronic diseases frequently observed in the Korean population and multiple genetic and epidemiological risk factors [20,21]. It includes residents from Ansan (an urban area) and Ansung (a rural area) in Gyeonggi Province, Korea. A baseline survey was conducted in 2001–2002, recruiting 10,030 adults aged 40–69 years, with follow-up surveys every 2 years. During follow-up, data on demographics, lifestyle habits, medical records, diagnostic records, metabolic markers, and disease prevalence were collected. The survey process was standardized and implemented by trained investigators [20]. This study utilized baseline and follow-up data up to 2020, excluding participants who met the following criteria: (1) missing >10 food items in the dietary assessment (*n* = 333); (2) male sex, as the study focused on menopausal status and osteoporosis risk in women (*n* = 4608); (3) osteoporosis diagnosis at baseline (*n* = 171); and (4) a total daily energy intake <500 kcal or >5000 kcal (*n* = 53). The final analysis included 4865 women, categorized into 2865 premenopausal and 2000 postmenopausal women (Figure 1). All participants provided written informed consent prior to their participation in the study [21,22]. The data collection and analysis procedures were approved by the Institutional Review Board (IRB) of the Korea Disease Control and Prevention Agency and Yeungnam University Ethics Committee (IRB number: 202112011-UE003).

### 2.2. Participants’ General Characteristics and Health Information

Demographic characteristics (age, residential area, education level, and household income level), lifestyle habits (smoking, alcohol consumption, and physical activity level), and disease information were collected through surveys [20]. Education level was classified into three categories: middle school graduation or lower, high school graduation, and college graduation or higher. Household income level was divided into four groups: <1 million South Korean won (KRW), 1–<2 million KRW, 2–<4 million KRW, and ≥4 million KRW per month. Smoking status was classified as current smoker or non-smoker (including both former and never smokers). Alcohol consumption, which was assessed by asking participants about their drinking habits, was classified as drinker (currently drinking) or non-drinker (previously or never drank). Physical activity was assessed using a questionnaire evaluating the type, duration, and frequency of physical activity, calculated in metabolic equivalents (METs [h/week]), and categorized into tertiles (low, medium, and high). Anthropometric measurements (i.e., height and weight) were performed by trained technicians, and body mass index (BMI) was calculated by dividing weight (kg) by the square of the height (m^2^). Menopausal status was determined using self-reported menstrual history. Women who reported ≥12 months of amenorrhea or provided an age at menopause were classified as postmenopausal, and all others were classified as premenopausal. The use of hormone therapy was categorized based on usage.

### 2.3. Dietary Assessment and Patterns

Dietary intake was evaluated using a semi-quantitative food frequency questionnaire (SQFFQ) with established validity and reproducibility, encompassing 103 items during the baseline survey (2001–2002) [23,24]. The consumption frequency of each food item was categorized into nine levels: “rarely,” “once a month,” “2–3 times a month,” “once a week,” “2–3 times a week,” “4–6 times a week,” “once a day,” “twice a day,” and “three times a day.” Portion sizes were classified into three categories: “half a serving,” “one serving,” and “one and a half servings.” Intake amount was calculated by multiplying consumption frequency by portion size.

To enable dietary pattern analysis, food items were aggregated into 23 food groups based on nutritional similarities and culinary uses, as described in previous studies [25]. Food groups reflected both the SQFFQ response items and examples included in the FFQ questions, as detailed in Supplementary Table S2. For example, milk, yogurt, and cheese were grouped under “milk and dairy products,” while protein-rich sources, such as legumes, nuts, and seafood, were categorized separately. Dietary patterns were identified through factor analysis using principal component analysis with varimax rotation, following the Kaiser criterion (eigenvalues > 1) and factor loadings > 0.310. To ensure interpretability, patterns, such as the “Vegetables and Seafood” and “Western” patterns, were named based on the food groups with the highest factor loadings.

### 2.4. Outcome Definition

Information on the history of osteoporosis was collected by trained investigators using a standardized questionnaire, which ascertained prior physician diagnosis, receipt of hospital treatment after diagnosis, any past use of osteoporosis medications, and current continuation of therapy. Specific drug names or classes were not collected. Self-reports were corroborated by medical record review when available [22].

Peripheral bone status was assessed using quantitative ultrasound (QUS) at the distal radius and mid-shaft tibia with a bone densitometer (Omnisense™ 7000s; Sunlight Medical, Ramat Gan, Israel). Following the device manual, landmarks were defined as the midpoint between the elbow and the tip of the middle finger for the distal radius, and the midpoint between the knee and the medial ankle for the tibia. At each site, three acquisitions were obtained and averaged. The device provided speed of sound and device-specific T- and Z-scores, which were derived from the manufacturer’s reference database [22].

The primary endpoint was a composite-defined osteoporosis (CDO) outcome, defined as meeting one or more of the following at follow-up: (i) self-reported physician diagnosis of osteoporosis; (ii) self-reported treatment or medication use for osteoporosis (including current therapy); or (iii) QUS-based low bone status, operationalized as a device T-score ≤−2.5 at either peripheral site [26]. Component-wise marginal counts for diagnosis, treatment/medication, and QUS low bone status—overall and stratified by menopausal status—are presented in Supplementary Table S1.

### 2.5. Statistical Analysis

Participants were classified by menopausal status and categorized into tertiles based on dietary patterns. Categorical variables, presented as frequencies and percentages, were compared using the chi-square test. Continuous variables, expressed as the mean ± standard error, were analyzed using general linear regression. The follow-up period for each participant was calculated from the baseline to the first occurrence of any component of the composite endpoint or to the last contact if no event occurred. Cox proportional hazards regression models were used to evaluate the association between dietary patterns and risk of CDO with hazard ratios (HRs) and 95% confidence intervals (CIs) reported. Confounders included in Models 1 and 2 were selected based on their established relationship with osteoporosis risk in prior studies [27,28,29] and preliminary analyses. Model 1 was adjusted for age, and Model 2 was further adjusted for education level, household income, smoking status, physical activity, BMI, total energy intake, and hormone therapy use. No significant effect modifiers were identified between dietary patterns and the risk of CDO, and the analysis was conducted accordingly. The linearity of the risk of CDO across dietary pattern tertiles was assessed by assigning median values of dietary patterns to each tertile and using these as continuous variables in regression analysis to calculate *P* for trend. This method was selected to account for the ordinal nature of tertiles and ensure a linear assessment of risk. All statistical analyses were conducted using SAS (version 9.4, Statistical Analysis System; SAS Institute Inc., Cary, NC, USA). Prior to analysis, data were preprocessed to handle missing values and ensure the accuracy of calculations. Missing dietary data were excluded based on pre-established criteria, and all variables were checked for normality and outliers to ensure a robust analysis. Statistical significance was set at α = 0.05.

## 3. Results

### 3.1. General Characteristics of Premenopausal and Postmenopausal Women at Baseline

Table 1 presents the baseline demographic and lifestyle characteristics of premenopausal and postmenopausal women. During the mean follow-up period of 8.26 years, 895 and 1525 cases of CDO were identified among premenopausal and postmenopausal women, respectively. The mean ages of premenopausal and postmenopausal women were 48.30 ± 0.15 and 58.32 ± 0.15 years, respectively. Regarding household income level, postmenopausal women had a larger proportion in the low-income category (57.85%) than premenopausal women (29.57%). Conversely, postmenopausal women had a smaller proportion in the high-income category (3.32%) than premenopausal women (7.53%). In terms of education, most premenopausal (57.89%) and postmenopausal (82.53%) women had a middle school education or lower. A relatively small proportion had a college education or higher, with 7.93% and 3.73% of premenopausal and postmenopausal women falling into this category, respectively. Regarding alcohol consumption, a higher percentage of postmenopausal women (80.17%) were non-drinkers than that of premenopausal women (70.23%). Contrariwise, 29.77% of premenopausal women were current drinkers compared with 19.83% of postmenopausal women. Most premenopausal (96.28%) and postmenopausal (96.11%) women were non-smokers. Physical activity levels, categorized into tertiles based on METs-h/week, demonstrated that a higher percentage of postmenopausal women (43.86%) were in the high physical activity category than that of premenopausal women (27.11%). The mean BMI values were 24.78 ± 0.06 and 25.17 ± 0.07 for premenopausal and postmenopausal women, respectively. The mean total energy intake for premenopausal and postmenopausal women was 1882.41 ± 11.73 and 1844.25 ± 14.43 kcal/day, respectively.

### 3.2. Factor Loading Matrix for the Factor Analysis of Food Groups in Premenopausal and Postmenopausal Women

Table 2 presents salient factor loadings (absolute value ≥ 0.310) from the factor analysis of food group intake patterns in premenopausal and postmenopausal women. The analysis identified three dietary patterns for each group. For premenopausal women, Factor 1 was labeled the “Vegetables and Seafood” pattern, which exhibited high factor loadings for potatoes, fresh vegetables, salted vegetables, mushroom, seaweed, fruits, legumes, fish and seafood, and salted seafood. Factor 2, designated the “Western” pattern, displayed high factor loadings for noodles, bread and rice cake, sweets, eggs, fish and seafood, red meat, poultry, ham, milk and dairy products, soft drinks and beverages, and coffee and tea. Factor 3 was defined as the “White rice, Meat, and Alcohol” pattern, with highly positive factor loadings for white rice, red meat, and alcohol and a highly negative factor loading for mixed rice.

For postmenopausal women, Factor 1 was labeled the “Diverse” pattern, which revealed high factor loadings for noodles, bread and rice cake, potatoes, fresh vegetables, mushroom, seaweed, fruits, eggs, fish and seafood, red meat, poultry, and ham. Factor 2, defined as the “Plant-Based” pattern, exhibited high factor loadings for mixed rice, potatoes, fresh vegetables, salted vegetables, fruits, and legumes. Factor 3, designated the “Sweets and Drinks” pattern, displayed high factor loadings for bread and rice cake, sweets, nuts, milk and dairy products, soft drinks and beverages, and coffee and tea. The complete factor loading matrix for all 23 food groups is provided in Supplementary Table S2.

### 3.3. Association Between Dietary Pattern and Composite-Defined Osteoporosis Incidence in Premenopausal and Postmenopausal Women

Table 3 summarizes the HRs and 95% CIs for the risk of CDO based on dietary patterns in premenopausal and postmenopausal women. In premenopausal women, the “Vegetables and Seafood” and “Western” patterns demonstrated no significant associations with the risk of CDO. However, the “White rice, Meat, and Alcohol” pattern indicated the greatest risk of CDO in the highest tertile (HR = 1.20, 95% CI: 1.01–1.43, *P* for trend = 0.04) after fully adjusting for age, education level, household income, smoking status, physical activity, BMI, total energy intake, and the use of hormone therapy. In postmenopausal women, the “Diverse” and “Plant-Based” patterns displayed no significant associations with the risk of CDO. The “Sweets and Drinks” pattern initially exhibited the lowest risk in the highest tertile in Model 1; however, this association was not significant after full adjustment (HR = 0.95, 95% CI: 0.82–1.10, *P* for trend = 0.5).

In sensitivity analyses using restricted cubic splines, the association between the “White rice, Meat, and Alcohol” pattern and CDO risk in premenopausal women appeared approximately linear, with no significant evidence of nonlinearity (*P* for nonlinearity = 0.59, Supplementary Figure S1).

## 4. Discussion

Using data from the KoGES cohort, we identified dietary patterns and their associations with the risk of CDO in premenopausal and postmenopausal women. In premenopausal women, the “Vegetables and Seafood,” “Western,” and “White rice, Meat, and Alcohol” patterns were identified. Among these, the “White rice, Meat, and Alcohol” pattern was significantly associated with an increased risk of CDO. In contrast, postmenopausal women exhibited the “Diverse,” “Plant-Based,” and “Sweets and Drinks” patterns, none of which demonstrated meaningful associations with the risk of CDO.

The significant association between the “White rice, Meat, and Alcohol” pattern and a higher risk of CDO in premenopausal women is broadly consistent with previous research. Studies have reported that diets high in alcohol, white rice, and red meat, coupled with low consumption of whole grains, are associated with adverse bone outcome [30,31,32,33,34]. For instance, a systematic review and meta-analysis of 31 studies from various regions, including Asia, Europe, and North America, found that “Western/Unhealthy” dietary patterns, characterized by high intakes of red meat, processed meat, refined grains, fat, sugar, sweets, and sodas, were significantly associated with a lower BMD and increased fracture risk [30]. In contrast, “Prudent/Healthy” dietary patterns, rich in fruits, vegetables, whole grains, and low-fat dairy, were associated with reduced risks [30]. Further supporting our findings, studies conducted in different countries, including Korea, China, the United States, Canada, and Sweden, have reported similar associations between dietary patterns and bone health [31]. A meta-analysis of 20 studies identified three common dietary patterns: “Healthy,” “Meat/Western,” and “Milk/Dairy.” The “Meat/Western” pattern was positively associated with a lower BMD and increased fracture risk. Additionally, a study assessing the dose–response relationship between alcohol consumption and bone health found a consistent increase in fracture risk with higher alcohol intake [34].

The higher risk of CDO in premenopausal women following the “White rice, Meat, and Alcohol” pattern can be explained by several mechanisms. Peak bone mass is typically reached before the age of 30, with rapid bone loss occurring after menopause owing to decreased estrogen levels [35,36]. Therefore, inappropriate premenopausal dietary habits potentially diminish peak bone mass, increasing the risk of fractures and osteoporosis [37]. Additionally, even with an adequate calcium intake, a diet high in dietary acids, such as refined grains, cheese, fish, and red meat, can negatively impact bone health [38,39,40]. These acidic foods can disrupt the body’s acid–base balance, leading to the release of calcium from bones to neutralize the acid, ultimately weakening bone structure [39,41]. Furthermore, high dietary inflammation indices, driven by proteins, cholesterol, carbohydrates, total fats, and saturated fatty acids, have been linked to an increased osteoporosis risk [42,43]. Osteoporosis involves chronic inflammatory responses where certain proinflammatory markers (e.g., tumor necrosis factor alpha, interleukin-1b, and interleukin-6) enhance osteoclast activity, reducing bone density [44,45,46]. Chronic excessive alcohol consumption also affects osteoblasts, crucial for bone formation, and decelerates bone turnover [10,47].

These findings suggest that dietary patterns high in alcohol, refined grains, and red meat can detrimentally affect bone health. While appropriate protein intake is essential for bone matrix formation and maintenance, and higher protein intake is associated with a reduced hip fracture risk [48,49,50], the negative impacts of the “White rice, Meat, and Alcohol” pattern on the risk of CDO potentially emanate from a combination of premenopausal dietary habits, dietary acid load, inflammatory food intake, and the alcohol-induced inhibition of bone formation.

The difference in significant associations between premenopausal and postmenopausal women is attributable to several factors. Menopause induces significant hormonal changes, particularly a decrease in estrogen, which serves an indispensable role in bone metabolism [51]. These alterations possibly overshadow the effects of dietary patterns on bone health among postmenopausal women, as estrogen deficiency accelerates bone resorption over bone formation, eliciting rapid bone loss that potentially masks the dietary effects observed in premenopausal women [52]. Additionally, premenopausal women are generally in a phase of maintaining or achieving peak bone mass, while postmenopausal women are experiencing bone loss [35]. Consequently, the impact of diet may be more pronounced in premenopausal women, as they are still building or maintaining bone density, whereas postmenopausal women may already experience reduced bone density owing to hormonal changes. Finally, unmeasured or inadequately measured confounders that differentially affect premenopausal and postmenopausal women may exist. Lifestyle factors, such as physical activity, socioeconomic status, and overall health status, may differentially interact with dietary patterns in these two groups. Further research is required to explore these differences and elucidate the complex interplay of diet, hormones, and bone health across different stages of a woman’s life.

This study has certain limitations. First, despite adjusting for potential confounders identified through literature review and preliminary analysis, residual confounding by factors not fully captured in the dataset—such as supplement use, medication history, or osteoporosis-related comorbidities—may still have existed. Our choice of covariates was guided by prior evidence and intended to balance adequate adjustment with the avoidance of over-controlling dietary pathways. Second, the findings are specific to Korean women and may not be generalizable to populations with different dietary habits. Third, osteoporosis was defined as a composite outcome including self-reported diagnosis, treatment/medication, and QUS-based low bone status. Because QUS is not equivalent to DXA-measured BMD, the endpoint should be interpreted as a screening proxy rather than a diagnostic reference standard. Still, QUS is portable, radiation-free, and suitable for large cohorts, justifying its use, though comparisons with DXA- or fracture-based studies should be made cautiously. In addition, fracture outcomes, the most clinically relevant endpoint, were not consistently collected and therefore could not be analyzed, which represents a limitation of this study. Fourth, Menopausal status was determined at baseline and used to stratify women into premenopausal and postmenopausal groups. Because some women initially classified as premenopausal may have transitioned during follow-up, this could have introduced nondifferential misclassification. Given that dietary exposures were also assessed at baseline only, our findings should be interpreted as reflecting baseline diet and menopausal status in relation to later risk of CDO. Finally, the SQFFQ used, although validated for the Korean diet, may exclude certain food items, potentially underestimating food intake. Moreover, the effect sizes observed were modest, and although statistically significant in some comparisons, their clinical relevance should be interpreted with caution.

## 5. Conclusions

In conclusion, premenopausal women following a dietary pattern high in alcohol, refined grains, and red meat (the “White rice, Meat, and Alcohol” pattern) exhibit a significantly greater risk of CDO. This emphasizes the pivotal role of dietary habits in bone health, specifically during the premenopausal period, when peak bone mass is established. Promoting dietary interventions that reduce the intake of refined grains, red meat, and alcohol may be a key strategy in preventing osteoporosis and maximizing bone health. These findings provide valuable insights into the relationship between diet and osteoporosis risk, reinforcing the development of targeted nutritional strategies that improve quality of life in women across all life stages.

## Figures and Tables

**Table 1:** Baseline characteristics of premenopausal and postmenopausal women.

Characteristics	Premenopausal Women (*n* = 2865)	Postmenopausal Women (*n* = 2000)
Age (years)	48.30 ± 0.15	58.32 ± 0.15
Household income		
Low	832 (29.57)	1131 (57.85)
Mid-low	877 (31.17)	477 (24.41)
Mid-high	893 (31.73)	282 (14.42)
High	212 (7.53)	65 (3.32)
Education		
Middle school graduation or lower	1650 (57.89)	1634 (82.53)
High school graduation	974 (34.18)	272 (13.74)
College graduation or higher	226 (7.93)	74 (3.73)
Alcohol consumption		
Non-drinkers	2003 (70.23)	1593 (80.17)
Current drinkers	849 (29.77)	394 (19.83)
Smoking		
Non-smokers	2714 (96.28)	1901 (96.11)
Current smokers	105 (3.72)	77 (3.89)
Physical activity ^1^		
Low	1040 (36.65)	569 (28.62)
Mid	1028 (36.24)	547 (27.52)
High	769 (27.11)	872 (43.86)
Body mass index (kg/m^2^)	24.78 ± 0.06	25.17 ± 0.07
Total energy intake (kcal/day)	1882.41 ± 11.73	1844.25 ± 14.43

Values are presented as the mean ± standard error or N (%). ^1^ Physical activity was categorized into tertiles based on the metabolic equivalent task-hours per week (METs-h/week).

**Table 2:** Factor loading matrix of food groups derived from dietary patterns in premenopausal and postmenopausal women.

Food Group	Premenopausal (*n* = 2865)			Postmenopausal (*n* = 2000)		
	Factor 1 (Vegetables & Seafood)	Factor 2 (Western)	Factor 3 (White Rice, Meat & Alcohol)	Factor 1 (Diverse)	Factor 2 (Plant-Based)	Factor 3 (Sweets & Drinks)
White rice			0.49			
Mixed rice			−0.35		0.41	
Noodles		0.44		0.50		
Bread and rice cake		0.55		0.40		0.39
Potatoes	0.39				0.43	
Sweets		0.47				0.6
Fresh vegetables	0.70			0.45	0.51	
Salted vegetables	0.53				0.54	
Mushroom	0.43			0.32		
Seaweed	0.51			0.41		
Fruits	0.43			0.33	0.37	
Legumes	0.60				0.65	
Eggs		0.36		0.42		
Nuts				0.03		0.46
Fish and seafood	0.57	0.41		0.63		
Salted seafood	0.40					
Red meat		0.50	0.33	0.69		
Poultry		0.50		0.58		
Ham/processed meat		0.52		0.49		
Milk and dairy products		0.41				0.55
Soft drinks/beverages		0.39				0.51
Coffee and tea		0.32				0.34
Alcohol			0.58			

Only loadings ≥0.310 are shown. The complete factor loading matrix for all 23 food groups is presented in Supplementary Table S2.

**Table 3:** Hazard ratios and 95% confidence intervals for composite-defined osteoporosis incidence across dietary pattern tertiles in premenopausal and postmenopausal women.

	Dietary Pattern Tertiles			*P* for Trend ^1^
	T1	T2	T3	
Premenopausal women (*n* = 2865)				
“Vegetables and Seafood” pattern				
No. of cases	280	295	320	
Model 1	ref	1.00 (0.85–1.18)	1.16 (0.98–1.36)	0.1
Model 2	ref	1.02 (0.85–1.22)	1.11 (0.91–1.35)	0.3
“Western” pattern				
No. of cases	359	272	264	
Model 1	ref	0.90 (0.76–1.06)	0.94 (0.79–1.11)	0.5
Model 2	ref	0.93 (0.78–1.11)	0.99 (0.81–1.20)	0.9
“White rice, Meat, and Alcohol” pattern				
No. of cases	285	316	294	
Model 1	ref	1.13 (0.96–1.33)	1.22 (1.03–1.44)	0.02
Model 2	ref	1.14 (0.96–1.36)	1.20 (1.01–1.43)	0.04
Postmenopausal women (*n* = 2000)				
“Diverse” pattern				
No. of cases	555	500	470	
Model 1	ref	0.86 (0.77–0.98)	0.88 (0.77–0.99)	0.1
Model 2	ref	0.87 (0.77–0.99)	0.95 (0.82–1.11)	0.6
“Plant-based” pattern				
No. of cases	489	502	534	
Model 1	ref	1.01 (0.89–1.15)	1.08 (0.96–1.22)	0.2
Model 2	ref	1.04 (0.91–1.19)	1.03 (0.89–1.19)	0.8
“Sweets and Drinks” pattern				
No. of cases	556	510	459	
Model 1	ref	0.92 (0.81–1.04)	0.79 (0.69–0.89)	<0.001
Model 2	ref	1.00 (0.88–1.14)	0.95 (0.82–1.10)	0.5

^1^ *P* for trend was evaluated by assigning the median value of each dietary intake tertile as a continuous variable. T, Tertile. Model 1: adjusted for age (continuous); Model 2: Additionally adjusted for education level (middle school graduation or lower, high school graduation, college graduation or higher), household income (low, mid-low, mid-high, high), smoking status (non-smoker, smoker), physical activity (continuous), body mass index (continuous), total energy intake (continuous) and the use of hormone therapy (use, not use).

## Data Availability

Restrictions apply to the availability of these data. Data was obtained from the Korean Genome and Epidemiology Study (KoGES) and are available from the National Institute of Health (NIH) at https://coda.nih.go.kr/common/procss/selectLttotProcss.do (accessed on 10 September 2025) with the permission of KoGES.

## Supplementary Materials

The following supporting information can be downloaded at: https://www.mdpi.com/article/10.3390/nu17182947/s1, Table S1. Counts by osteoporosis diagnosis, treatment/medication, and QUS low bone status and the osteoporosis composite, overall and by menopausal status; Table S2. Complete factor loading matrix of 23 food groups and their definitions in premenopausal and postmenopausal women; Figure S1. Hazard ratios and 95% confidence intervals for the nonlinear relationship between the “White rice, Meat, and Alcohol” pattern and composite osteoporosis in premenopausal women, evaluated using restricted cubic splines (*P* for nonlinearity = 0.59).
