# Supplementary material for: Dietary Patterns and the Risk of Composite-Defined Osteoporosis in Pre- and Postmenopausal Women: A Prospective Cohort Study
Source: Nutrients. 2025 Sep 12;17(18):2947. doi: 10.3390/nu17182947 (PMC12473033; doi:10.3390/nu17182947)
Supplement: Supplementary file 1 [file nutrients-17-02947-s001.zip › nutrients-3863485-supplementary.pdf]

Supplementary File

Dietary Patterns and the Risk of Osteoporosis in Pre- and Postmenopausal Women: A Prospective Cohort Study

Supplementary Table S1. Counts by osteoporosis diagnosis, treatment/medication, and QUS low bone status and the osteoporosis composite, overall and by menopausal status

Group	Participants, n	Composite outcome, n (%)	Physician diagnosis, n (%)	Treatment/medication, n (%)	QUS low bone, n(%)
Overall	4,865	2,424 (49.8)	1,792 (36.9)	861 (17.7)	1,085 (22.3)
Premenopausal	2,865	897 (31.3)	589 (20.6)	283 (9.9)	373 (13.0)
Postmenopausal	2,000	1,527 (76.4)	1,203 (60.2)	577 (28.9)	712 (35.6)

Notes: Counts are marginal and not mutually exclusive; individuals may meet multiple components, so the composite ≤ sum of components. *QUS low bone*: device T-score ≤ −2.5 at distal radius or mid-shaft tibia.

Supplementary Table S2. Complete factor loading matrix of 23 food groups and their definitions in premenopausal and postmenopausal women

Food Group	Item included	Premenopausal			Postmenopausal		
		Factor 1 (Vegetables & Seafood)	Factor 2 (Western)	Factor 3 (Rice, Meat & Alcohol)	Factor 1 (Diverse)	Factor 2 (Plant-based)	Factor 3 (Sweets & Drinks)
White rice	White rice	0.12	−0.09	**0.49**	**0.69**	−0.12	0.11
Mixed rice	Barley rice, mixed grain rice	0.28	−0.19	−0.35	−0.15	**0.41**	0.13
Noodles	Instant noodles, wheat noodles, black bean noodles (jjajangmyeon), buckwheat/cold noodles (naengmyeon), glass noodles (dangmyeon)/japchae	0.26	**0.44**	0.02	**0.5**	0.09	−0.04
Bread and rice cake	Grain powder drink (misutgaru), white bread, sweet red bean bun, other bread, pizza, white rice cake/tteokguk cake, baekseolgi, injeolmi	0.12	**0.55**	−0.29	**0.4**	−0.07	**0.39**
Potatoes	Potato, sweet potato	**0.39**	0.12	−0.27	0.29	**0.43**	0.04
Sweets	Choco pie/cake, cookies/crackers, candy/chocolate	−0.04	**0.47**	−0.27	−0.07	0.07	**0.6**
Fresh vegetables	Green chili pepper, chili leaves, spinach, lettuce, perilla leaves, chives/water dropwort, other green leafy vegetables, radish, balloon flower root/deodeok, onion, cabbage, cucumber, bean sprouts/mung bean sprouts, carrot, pumpkin, zucchini, vegetable juice, bracken/sweet potato stems	**0.7**	0.26	0	**0.45**	**0.51**	0.19
Salted vegetables	Napa kimchi, cubed radish kimchi, watery kimchi (nabak kimchi/dongchimi), other kimchi, pickled vegetables (jangajji)	**0.53**	−0.14	0.06	0.14	**0.54**	−0.07
Mushroom	Oyster mushroom, other mushrooms	**0.43**	0.24	0.05	**0.32**	0.09	**0.31**
Seaweed	Laver (gim), kelp/sea mustard (dashima/miyeok)	**0.51**	0.24	0.01	**0.41**	**0.3**	0.24

Fruits	Persimmon/dried persimmon, mandarin orange, melon, banana, pear, apple, orange, watermelon, peach/plum, strawberry, grape, tomato	**0.43**	0.17	0.1	**0.33**	**0.37**	0.09
Legumes	Soybeans, tofu, soybean paste (doenjang)/fermented soybean (cheonggukjang), soy milk	**0.6**	−0.14	−0.27	−0.01	**0.65**	0.12
Eggs	Chicken egg, quail egg	0.24	**0.36**	−0.09	**0.42**	0.22	0.21
Nuts	Peanut, almond, pine nut	0.11	0.27	−0.08	0.03	0.05	**0.46**
Fish and seafood	Raw fish (sashimi), hairtail, eel, croaker, pollock/dried pollock, oily fish (e.g., mackerel), anchovy, canned tuna, fish cake/crab stick, crab, clam, oyster, shrimp, squid/octopus	**0.57**	**0.41**	0.13	**0.63**	0.2	0.27
Salted seafood	Fermented salted seafood (jeotgal)	**0.4**	0.06	0.03	0.26	0.23	0.09
Red meat	Pork, beef, organ meats	**0.31**	**0.5**	**0.33**	**0.69**	−0.12	0.11
Poultry	Chicken	0.18	**0.5**	0.29	**0.58**	−0.12	−0.11
Ham/processed meat	Ham and sausages	−0.02	**0.52**	0.05	**0.49**	−0.25	0.18
Milk and dairy products	Milk, yogurt, ice cream, cheese	0.18	**0.41**	−0.24	0.2	0.01	**0.55**
Soft drinks/beverages	Carbonated drinks, other soft drinks	0.07	**0.39**	0.15	0.11	0.04	**0.51**
Coffee and tea	Coffee, tea (e.g., green tea), and additives to coffee/tea (sugar, creamer)	−0.02	**0.32**	0.11	0.09	−0.18	**0.34**
Alcohol	Makgeolli, beer, sake/cheongju, wine, soju, whiskey	0.02	0.05	**0.58**	0.11	−0.20	0.1

Food items reflect both the FFQ items and examples included in the FFQ questions

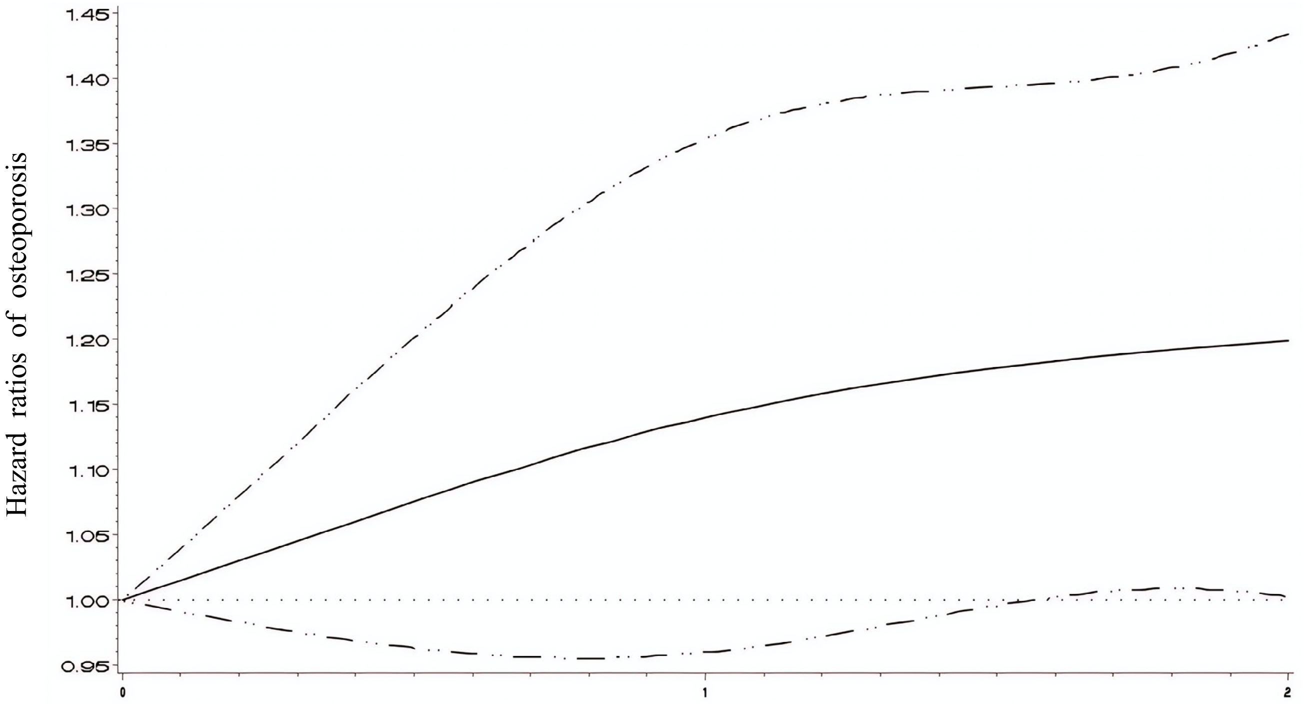

Supplementary Figure S1. Hazard ratios and 95% confidence intervals for the nonlinear relationship between the "White rice, Meat, and Alcohol" pattern and composite osteoporosis in premenopausal women, evaluated using restricted cubic splines (P for nonlinearity = 0.59). The model was adjusted for age, education level, household income, smoking status, body mass index, physical activity, total energy intake, and hormone use.
